# Immunogenicity and safety of the first indigenously developed Indian tetravalent influenza vaccine (split virion) in healthy adults ≥ 18 years of age: A randomized, multicenter, phase II / III clinical trial

**DOI:** 10.1080/21645515.2018.1441654

**Published:** 2018-03-21

**Authors:** Shrikant Sharma, Veer Bahadur Singh, Sanjay Kumar, Vipul Prajapati, Jitendra Patel, Rajesh Vukkala, Sanjay Kumar Jangid, Jayesh Sanmukhani, Gaurav Gupta, Pradip Patel, Ravindra Mittal, Reinhard Glueck

**Affiliations:** a Assistant Professor, Department of Medicine, SMS Medical College & Hospital, Jaipur, India; b Senior Professor, Department of Medicine, P.B.M. Hospital & S.P. Medical College, Bikaner, India; c Consultant Physician M.V. Hospital and Research Centre, Lucknow, India; d Assistant Professor, Department of Medicine, GCS Medical College, Hospital and Research Centre, Ahmedabad, India; e Consultant Physician, Medilink Hospital, Ahmedabad, India; f Consultant Physician, Indo-US Superspeciality Hospital, Hyderabad, India; g Assistant Professor, Department of Medicine, Hi-Tech Medical College & Hospital, Bhubaneswar, India; h Senior Manager, Department of Clinical Research and Regulatory Affairs, Cadila Healthcare Limited, Ahmedabad, India; i Deputy General Manager, Department of Virology and Biotechnology, Vaccine Technology Centre, Cadila Healthcare Limited, Ahmedabad, India; j HEAD, Operations (Vaccines), Cadila Healthcare Limited, Ahmedabad, India; k Senior Vice President, Department of Clinical Research and Regulatory Affairs, Cadila Healthcare Limited, Ahmedabad, India; l Chief Scientific Officer, Vaccine Technology Centre, Cadila Healthcare Limited, Ahmedabad, India

**Keywords:** Tetravalent Influenza vaccine, Trivalent Influenza vaccine, immunogenicity, Cadila Healthcare Limited, Vaxigrip, Sanofi Pasteur

## Abstract

This phase II / III clinical trial was conducted to evaluate the immunogenicity and safety of the Tetravalent Influenza vaccine (Split virion) I.P. (TetIV) developed indigenously in the country for the first time by M/s Cadila Healthcare Limited, India containing two influenza A and two influenza B strains, one of each, Yamagata (B/Phuket) and Victoria (B/Brisbane) lineage and also compare it to that of an licensed seasonal Trivalent Influenza vaccine (TriIV) of Sanofi Pasteur India Private Limited, containing the two influenza A and only the Yamagata lineage (B/Phuket) strain. Three hundred and fifty subjects of either sex, aged more than 18 years of age, were randomized in a 1:1 ratio to receive either the TetIV or TriIV. Immunogenicity assessments (antibody against A/H1N1, A/H3N2, B/Phuket and B/Brisbane) were done by Haemagglutination Inhibition assay at baseline and 21 d after vaccination. Solicited (local and systemic) and unsolicited adverse events were recorded for up to 42 d following vaccination. The TetIV was found to fulfill the criteria set by the European and the US regulatory authorities and WHO guidance on the requirements of clinical data for licensure of seasonal inactivated influenza vaccines. The seroconversion rates with TetIV were 93.5% for A/H1N1, 90.0% for A/H3N2, 70.0% for B/Phuket and 82.9% for B/Brisbane strain. There was no significant difference in the seroconversion and seroprotection rates at day 21 for A/H1N1, A/H3N2 and B/Phuket in the two groups while the TetIV was superior to the TrivIV for the seroconversion and the seroprotection rate for the B/Brisbane strain (Victoria lineage). Both the vaccines were well tolerated by all the study participants; addition of the fourth strain in the TetIV did not compromise the safety as compared to TriIV. The most common systemic adverse event reported in both the groups was headache followed by fever.

## Introduction

Influenza is a highly infectious respiratory disease affecting all the age groups with the most vulnerable populations being the young children, the elderly and individuals with chronic diseases.^1^ It is a major cause of hospitalization and deaths in the elderly population and those with chronic illnesses. Among the healthy adults, influenza is an important cause of outpatient medical visits and worker absenteeism, burdening health care systems and leading to substantial societal costs.^2^

Although new antivirals agents have improved the ability to treat influenza infection, the most effective means for reducing the number of influenza cases is the annual vaccination. Vaccine effectiveness depends on the similarity between the virus strains present in the vaccine and those circulating in the community. Trivalent influenza vaccines are traditionally composed of one A/H1N1 strain, one A/H3N2 strain, and one B strain, each chosen to provide protection against the strains anticipated to circulate during the upcoming influenza season. Two distinct lineages of influenza B (the Victoria and Yamagata lineages) have co-circulated worldwide since 2000; neither providing good cross-protection against the other.^3^ Unfortunately, the ability to predict with acceptable accuracy which B lineage will be dominant in an upcoming season has been unsatisfactory, with frequent mismatches. The inclusion of an influenza B strain from both the Victoria and Yamagata lineages in a quadrivalent / tetravalent vaccine could improve protection against influenza B, and could reduce the burden of seasonal influenza illness, hospitalization, and death.^4^ As such, for the first time, the World Health Organization (WHO) recommended B strains from both lineages for use in vaccines for the 2012–2013 season in the Northern Hemisphere.^5^ Following the WHO recommendation, various vaccine manufacturers have developed quadrivalent / tetravalent influenza vaccines but none of these vaccines is approved and marketed in India.

Considering the WHO recommendation and the need of a better influenza vaccine in India, M/s Cadila Healthcare Limited has developed a Tetravalent Influenza vaccine (Split virion) I.P. (TetIV), containing two A strains (H1N1 & H3N2) and two B strains (Yamagata & Victoria) for the first time in the country. The vaccine has been shown to be safe in preclinical animal toxicity studies and in phase I study in adult subjects.^6^ This phase II / III clinical trial was conducted in adult healthy subjects of either sex to evaluate the immunogenicity and safety of TetIV of M/s Cadila Healthcare Limited for fulfillment of serologic parameters required for influenza vaccines and also compare them with that of another licensed seasonal Trivalent Influenza vaccine (Vaxigrip, Sanofi Pasteur India Private Limited) (TriIV).

## Results

**Figure 1.**
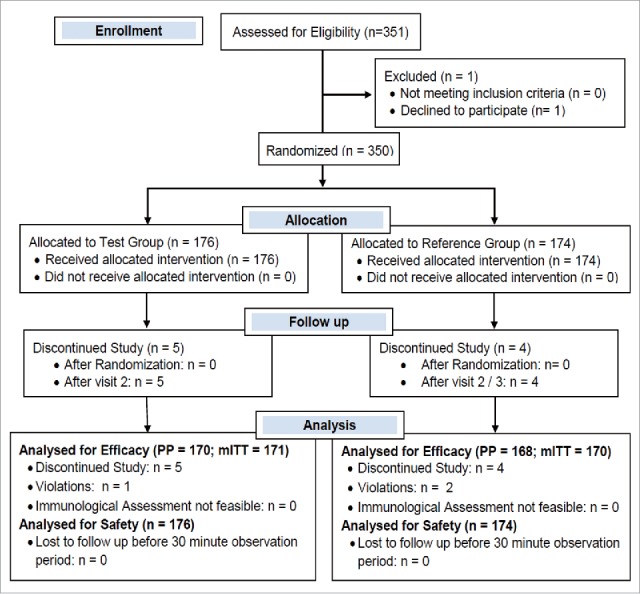
Flow of subjects in the study.

Three hundred and fifty subjects were enrolled in this randomized, single blind, active controlled, multicenter phase II / III clinical trial. One hundred and seventy six subjects were assigned to the TetIV group and 174 subjects to the TriIV group. All the 176 subjects of TetIV group completed their post vaccination 30-minute observation period and were thus considered for safety analysis. Six subjects in the TetIV group did not complete the study as per the protocol (5 lost to follow up, 1 violation) and hence, 170 subjects were considered for the per protocol immunogenicity analysis. Among the 174 subjects in the TriIV group, 168 were considered for the per protocol immunogenicity analysis (4 lost to follow-up; 2 violations), and all 174 were considered for safety analysis. The flow of subjects through the study is shown in Fig. 1. The demographic and baseline characteristics of the subjects are shown in Table 1.

**Table 1.** Demographic and baseline characteristics of the enrolled subjects.

	TetIV Group (N = 176)	TriIV Group (N = 174)
Age (Years)	45.7 ± 19.4	44.8 ± 18.6
Sex*		
Male	99 (56.2%)	108 (62.1%)
Female	77 (43.8%)	66 (37.9%)
Height (cm)	161.8 ± 8.0	163.1 ± 9.5
Weight (kg)	64.2 ± 13.2	65.0 ± 13.8
BMI	24.4 ± 4.4	24.3 ± 4.3

Data expressed as mean ± SD.

*Data expressed as n (%).

### Immunogenicity

Table 2 presents the primary immunogenicity end point data i.e. the seroconversion rate and the seroprotection rate for all the four strains at day 21 in the TetIV group. The lower bound of the 95% CI of the seroconversion rate and the seroprotection rate was greater than the recommended limit of 40% and 70%, respectively for all the four strains in the TetIV group, thus fulfilling the primary criteria as adapted from the Committee for Medicinal Products for Human Use (CHMP),^7^ US Food and Drug Administration (US FDA)^8^ and World health Organization (WHO)^9^ guidance on the requirements of clinical data for licensure of seasonal inactivated influenza vaccines.

**Table 2.** Seroconversion rate and Seroprotection rate with TetIV of M/s Cadila Healthcare Limited 21 days after vaccination (N = 170; PP Population).

Viral Strain	Seroconversion rate	Seroprotection rate
A/H1N1	159 (93.5%) (88.7% to 96.7%)	167 (98.2%) (94.9% to 99.6%)
A/H3N2	153 (90.0%) (84.5% to 94.1%)	167 (98.2%) (94.9% to 99.6%)
B/Phuket	119 (70.0%) (62.5% to 76.8%)	145 (85.3%) (79.1% to 90.3%)
B/Brisbane	141 (82.9%) (76.4% to 88.3%)	166 (97.7%) (94.1% to 99.4%)

Data expressed as n (%) (95% CI).

The comparison of the seroconversion rate, the seroprotection rate and the geometric mean titers (GMT) in the two groups is shown in Table 3. There was no significant difference in the seroconversion and the seroprotection rates at day 21 for A/H1N1, A/H3N2 and B/Phuket in the two groups. The GMT at day 21 after vaccination for A/H1N1 was superior in the TetIV group as compared to the TriIV group while there was no significant difference in the GMTs for A/H3N2 and B/Phuket in the two groups. The TetIV was superior to the TriIV for all the three immunogenicity parameters i.e. the seroconversion rate, the seroprotection rate and the GMT at day 21 after vaccination for the B/Brisbane strain (Victoria lineage). This was due to the presence of the additional B/Brisbane strain in the TetIV as compared to the TriIV.

**Table 3.** Seroconversion rate, Seroprotection rate and GMTs in the two groups 21 days after vaccination.

	Immunogenicity end point	TetIV Group (N = 170)	TriIV Group (N = 168)	P value
A/H1N1	Seroconversion^*^	93.5% (88.7% – 96.7%)	88.7% (82.9% – 93.1%)	0.17
	Seroprotection^*^	98.2% (94.9% – 99.6%)	94.1% (89.3% – 97.1%)	0.09
	GMT (baseline)^#^	22.2 (18.2 – 27.2)	24.4 (20.2 – 29.4)	0.51
	GMT (day 21)^#^	637.4 (532.4 – 763.1)	320.0 (260.8 – 392.7)	<0.01
A/H3N2	Seroconversion^*^	90.0% (84.5% – 94.1%)	89.3% (83.6% – 93.5%)	0.97
	Seroprotection^*^	98.2% (94.9% – 99.6%)	96.4% (92.4% – 98.7%)	0.49
	GMT (baseline)^#^	37.8 (30.9 – 46.2)	31.5 (25.8 – 38.4)	0.20
	GMT (day 21)^#^	732.2 (604.2 – 886.4)	591.8 (484.5 – 722.7)	0.13
B/Phuket	Seroconversion^*^	70.0% (62.5% – 76.8%)	72.6% (65.2% – 79.2%)	0.68
	Seroprotection^*^	85.3% (79.1% – 90.3%)	87.5% (81.5% –92.1%)	0.67
	GMT (baseline)^#^	18.3 (15.5 – 21.5)	15.7 (13.5 – 18.4)	0.19
	GMT (day 21)^#^	105.6 (88.4 – 126.1)	136.8 (111.5 – 167.8)	0.06
B/Brisbane	Seroconversion^*^	82.9% (76.4% – 88.3%)	55.4% (47.5% – 63.0%)	<0.01
	Seroprotection^*^	97.7% (94.1% – 99.4%)	82.1% (75.5% – 87.6%)	<0.01
	GMT (baseline)^#^	25.0 (21.0 – 29.8)	22.8 (19.2 – 27.2)	0.46
	GMT (day 21)^#^	203.5 (170.8 – 242.6)	84.8 (70.5 – 101.8)	<0.01

* Data presented as % (95%CI).

# Data presented as mean (95%CI).

Table 4 presents the age stratified immunogenicity data in the adults and the elderly. The lower bound of the 95% CI of the seroconversion rate and the seroprotection rate was greater than the recommended limit of 40% & 70%, respectively for the adult cohort and 30% & 60%, respectively for the elderly cohort for all the four strains in the TetIV group, thus fulfilling the criteria for licensure of the seasonal inactivated influenza vaccines.

**Table 4.** Sub group analysis of immunogenicity data in the two groups 21 days after vaccination.

	Adults: ≥18 to 60 years		Elderly: >60 years	
	TetIV Group (N = 111)	TriIV Group (N = 112)	TetIV Group (N = 59)	TriIV Group (N = 56)
A/H1N1				
Seroconversion^*^	94.6% (88.6% – 98.0%)	87.5% (80.0% – 93.0%)	91.5% (81.3% – 97.2%	91.1% (80.4% – 97.0%)
Seroprotection^*^	99.1% (95.1% – 100.0%)	94.6% (88.7% – 98.0%)	96.6% (88.3% – 99.6%)	92.9% (82.7% – 98.0%)
GMT (baseline)^#^	25.0 (19.3 – 32.5)	27.9 (21.9 – 35.6)	17.8 (12.9 – 24.5)	18.6 (14.2 – 24.2)
GMT (day 21)^#^	711.7 (586.4 – 863.7)	355.5 (277.2 – 456.0)	518.0 (357.0 – 751.7)	259.3 (180.1 – 373.2)
A/H3N2				
Seroconversion^*^	88.3% (80.8% – 93.6%)	89.3% (82.0% – 94.3%)	93.2% (83.5% – 98.1%)	89.3% (78.1% – 96.0%)
Seroprotection^*^	99.1% (95.1% – 100.0%)	98.2% (93.7% – 99.8%)	96.6% (88.3% – 99.6%)	92.9% (82.7% – 98.0%)
GMT (baseline)^#^	38.1 (29.7 – 48.8)	33.0 (25.7 – 42.4)	37.3 (26.3 – 52.9)	28.6 (20.6 – 39.8)
GMT (day 21)^#^	660.3 (534.6 – 815.6)	648.0 (519.8 – 807.7)	889.3 (604.6 – 1308.1)	493.5 (326.3 – 746.3)
B/Phuket				
Seroconversion^*^	73.9% (64.7% – 81.8%)	76.8% (67.9% – 84.2%)	62.7% (49.2% – 75.0%)	64.3% (50.4% – 76.7%)
Seroprotection^*^	91.0% (84.1% – 95.6%)	92.0% (85.3% – 96.3%)	74.6% (61.6% – 85.09%)	78.6% (65.6% – 88.4%)
GMT (baseline)^#^	21.8 (17.9 – 26.6)	16.5 (13.7 – 19.9)	13.1 (10.0 – 17.2)	14.3 (10.7 – 19.1)
GMT (day 21)^#^	123.1 (100.7 – 150.4)	167.1 (131.1 – 212.9)	79.1 (56.1 – 111.4)	91.7 (63.6 – 132.1)
B/Brisbane				
Seroconversion^*^	82.9% (74.6% – 89.4%)	58.0% (48.3% – 67.3%)	83.1% (71.0% – 91.6%)	50.0% (36.3% – 63.7%)
Seroprotection^*^	100.0% (96.7% – 100.0%)	88.4% (81.0% – 93.7%)	93.2% (83.5% – 98.1%)	69.6% (55.9% – 81.2%)
GMT (baseline)^#^	28.9 (23.9 – 35.9)	26.9 (21.6 – 33.5)	19.1 (14.2 – 25.6)	16.4 (12.4 – 21.7)
GMT (day 21)^#^	228.4 (188.6 – 276.7)	105.7 (84.8 – 131.7)	163.8 (114.7 – 234.0)	54.5 (40.0 – 74.2)

* Data presented as % (95%CI).

# Data presented as mean (95% CI).

### Safety

**Figure 2.**
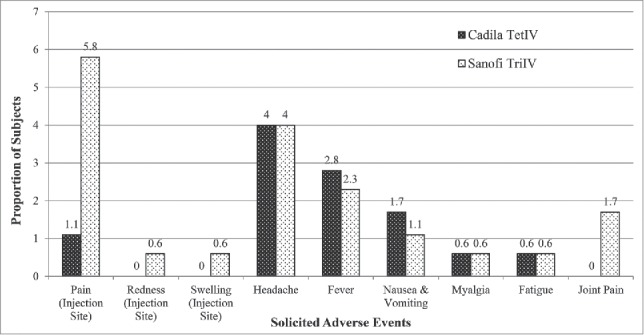
Adverse events reported post influenza vaccination.

Twenty six adverse events were reported in 20 subjects in the TetIV group (11.4% adverse event rate), and 39 adverse events were reported in 27 subjects in the TriIV group (15.5% adverse event rate). The most common local adverse event reported during the study was pain at the site of injection and the most common systemic adverse event reported during the study was headache followed by fever and nausea & vomiting in both the groups. The details of the solicited adverse events reported during the study are shown in Fig. 2. There was no difference in the adverse event profile between the two groups (*P*>0.05). The unsolicited adverse events reported during the study included upper respiratory tract infection (1.7%), vertigo (1.7%) and body ache (0.6%) in the TetIV group and upper respiratory tract infection (2.9%), vertigo (1.1%), joint pain (1.7%), back pain (0.6%) and weakness (0.6%) in the TriIV group.

Twenty five of the 26 adverse events in the TetIV group and 35 of the 38 adverse events reported in the TriIV group were “mild” in severity. Most of the adverse events lasted for 1–3 d (96.2% in the TetIV group, and 79.5% in the TrivIV group). All the adverse events resolved completely, with or without symptomatic treatment, during the study period. No “serious” or “severe” adverse events were reported in the subjects during the study.

Subgroup analysis of safety data showed that there was no difference in the adverse events reported in the adults and the elderly subjects during the study. Among the 113 adult subjects (>18 – 60 years) in the TetIV group, 13 subjects had 18 adverse events (adverse event rate of 11.5%) while among the 63 elderly subjects, 7 subjects had 8 adverse events (adverse event rate of 11.1%). The most common adverse event reported in the elderly subjects was headache.

## Discussion

This study presents the results of the phase II/III clinical trial conducted to assess the immunogenicity and safety of tetravalent influenza vaccine of M/s Cadila Healthcare Limited in Indian subjects aged ≥ 18 years, the first such vaccine to have been developed indigenously in the country. The study showed that the new vaccine was able to elicit a strong Haemagglutination Inhibition (HI) antibody response against all the four strains i.e. A/H1N1, A/H3/N2, B/Yamagata (B/Phuket) & B/Victoria (B/Brisbane) and fulfilled the immunogenicity criteria as adapted from the CHMP, US FDA and WHO guidance on the requirements of clinical data for licensure of seasonal inactivated influenza vaccines. The TetIV had superior antibody response for the additional B strain (B/Victoria) compared to the TriIV in which the strain was absent. When a new strain is added to a vaccine, it is important to ensure that the new antigen does not interfere with the immune response to the existing vaccine antigens. For the TetIV in our study, the absence of this interference was established by demonstrating comparability between the HI antibody responses elicited by the TetIV and the approved TriIV for the three shared strains; there was no significant difference in the seroconversion rates & the seroprotection rates to the three shared strains and in the GMTs against the two shared strains (A/H3N2 and B/Phuket) in the two vaccines. The higher GMT response against A/H1N1 with the TetIV could be attributed to the variation in the antigenic content between the two vaccine formulations.

Although influenza B causes disease in all the age groups, it is believed to have a higher incidence rate in the older children and young adults while influenza A is reported to be associated with higher rates of influenza related complications and deaths in the elderly.^10^ Nonetheless, influenza B is responsible for epidemics in adults every 2–4 years and infection with influenza B virus confers an important risk of severe illness and hospitalization.^10^^,^^11^ Based on the burden of influenza B and the inability to accurately predict which influenza B lineage will circulate, it is pertinent that addition of both the lineages of the influenza B strains in the seasonal influenza vaccines will improve their effectiveness. Further, this provides a direct benefit to the vaccine recipients whenever the circulating influenza B virus(es) does not match the lineage chosen for the trivalent vaccines, either because the lineage prediction was incorrect or because both lineages co-circulated to a significant degree. Moreover, in seasons in which influenza B circulation is minimal or B viruses are well matched to the trivalent vaccine strain, vaccination with a tetravalent influenza vaccine still provides benefit to the individual by increasing immunity to both the lineages of influenza B, with potential clinical benefit in subsequent seasons. This accumulated immunity has more relevance for influenza B than for influenza A because antigenic drift is more limited with influenza B viruses.^11^

The seroconversion rates with the TetIV day 21 after the vaccination were 93.5%, 90.0%, 70.0% and 82.9% for A/H1N1, A/H3N2, B/Yamagata and B/Victoria, respectively, showing that the immune response against influenza A strains is stronger than the influenza B strains. These results are better than the results of the other tetravalent / quadrivalent influenza vaccines approved and marketed in other countries. In one of the Phase III clinical trials with quadrivalent influenza vaccine manufactured by GlaxoSmithKline (GSK), Dresden, Germany, the seroconversion rates for A/H1N1, A/H3N2, B/Yamagata and B/Victoria were 77.5%, 71.5%, 58.1% and 61.7%, respectively.^10^ In another Phase III clinical trial conducted with quadrivalent influenza vaccine manufactured by Sanofi Pasteur (Swiftwater, PA, USA), the seroconversion rates for the four strains as mentioned above were 69%, 79%, 40% and 71%, respectively.^12^ The better immune response in the current study could be attributed to the ethnic differences in the population studied. This is the first study showing the immunogenicity of Trivalent and Tetravalent Influenza vaccines in the Indian population and hence no historical data in the local population is available for comparison.

It was noted in our study that besides the good HI antibody titers against the three shared strains in the TriIV, it also showed seroconversion (55.4%) and seroprotection (82.1%) against the unshared B/Brisbane strain (B/Victoria lineage), although these were significantly lower than that with the TetIV. These results are in line with those seen in previous trials wherein the seroconversion and the seroprotection rates were 47.5% and 96.1% respectively for the B/Victoria lineage with Trivalent vaccine containing B/Yamagata strain and the results for B/Yamagata were 45.6% and 97.9% with the Trivalent vaccine containing the B/Victoria strain.^10^ This booster response to the B lineage absent from the trivalent influenza vaccine is likely to be due to high baseline antibody levels (66 of the 168 subjects i.e. 39.3% had antibody level ≥40 at baseline, data not shown in results section). Moreover, as this trial was conducted from October 2015 to February 2016 which was peak season for influenza in India, environmental exposure and subclinical infection could also have contributed to rise in antibody titres against the B strain absent from the vaccine. Nevertheless, these cross reactive antibodies do not co-relate with protection, as very high rates of infection have been observed in the elderly population exposed to vaccine-lineage mismatched influenza B viruses even when the vaccine had elicited cross-reactive booster response.^10^^,^^13^

One of the priority groups for influenza vaccination is the elderly population with age more than 60 years. Also, it has also been known that the elderly population has diminished response to influenza vaccines.^12^^,^^14^ The age stratified data in our study showed that the HI antibody response appeared to decrease with advancing age. The GMT for all the 4 strains was higher in subjects age 18–60 years as compared to >60 years of age (except for GMT of A/H3N2 in TetIV group). Nevertheless, serological assessments for both the vaccines in the study met the CHMP criteria for each strain for both the age groups. Similar reduction in response (seroconversion rate and GMT) in the elderly population has been seen in previous studies with the various trivalent and tetravalent influenza vaccines.^10^^,^^12^^,^^15^ In one such study with tetravalent influenza vaccine, the post vaccination GMT in the elderly population (≥ 61 years) was 118, 305, 78.3 and 155 for A/H1N1, A, H3N2, B1 and B2 respectively; while the corresponding GMTs in the adult population (18-60 years) were 221, 289, 132 and 210, respectively.^12^

The TetIV contained 60 µg influenza antigens while the TriIV contained 45 µg of influenza antigens (15µg of each antigen). The higher antigenic content in the TetIV could have led to higher reactogenicity and adverse events, however the adverse event profile of both the vaccines was found to be similar in the study. Moreover, there was no significant difference in the adverse event profile in the elderly subjects (> 60 years of age) as compared to that in the adult subjects (18–60 years). These results suggest that addition of the fourth strain in the TetIV did not compromise the safety as compared to the TriIV. The safety profile of the TetIV of M/s Cadila Healthcare Limited was similar to the safety profile of the other approved and marketed tetravalent / quadrivalent influenza vaccines internationally.^16^^,^^17^

Although there are several published clinical trials demonstrating the immunogenicity and safety of tetravalent and trivalent influenza vaccines, to the best of our knowledge, this is the first report establishing the immune response and safety of any multivalent (Trivalent / Tetravalent) split virion vaccine in the Indian population. Although trivalent influenza vaccines have been marketed in the country since many years, there is no data on the immunogenicity and safety of these vaccines in Indian population in the public domain. This study has given an insight in the response of the multivalent influenza vaccines in the Indian population. Also, this is the first tetravalent influenza vaccine to have received marketing authorization in the country and is also the first tetravalent influenza vaccine manufactured indigenously in the country. Until now all the inactivated trivalent influenza vaccines being marketed in India were imported from other countries. Local manufacturing of the vaccine will help reduce cost and increase the usage thereby promoting public health in the region and will also ensure preparedness in case of pandemics.

One of the limitations of the study is that it was only a serological study and further efficacy studies would be required to evaluate the impact of including both the lineages of influenza type B strain in reducing the burden of the influenza disease in the community. Another limitation of the study was its single blind design which could have had some bias in the reporting of the adverse events. A double blind study could not be conducted as there was a difference in the packaging of the two vaccines (TetIV in a vial and TriIV in a pre-filled syringe) and a double dummy design would have led to administration of two injections in all the participants which could have raised ethical concerns.

## Conclusion

The results of this randomized, single blind, multicentric, phase II / III clinical trial show that the Inactivated Tetravalent Influenza vaccine (split virion) I.P. developed indigenously in the country for the first time by M/s Cadila Healthcare Limited, India provides superior immunogenicity for the additional B strain (B/Brisbane, Victoria lineage) as compared to the marketed Inactivated Trivalent Influenza vaccine of M/s Sanofi Pasteur India Private Limited, without interfering with the antibody responses to the other three antigens in the Trivalent Influenza vaccine. Moreover, the Inactivated Tetravalent Influenza vaccine (split virion) I.P. of M/s Cadila Healthcare Limited had a similar safety profile as that of the marketed Inactivated Trivalent Influenza vaccine of M/s Sanofi Pasteur India Private Limited.

## Material and methods

This prospective, randomized, single blind, parallel group, active controlled, multicenter, phase II / III clinical trial was conducted at 7 tertiary care centers in India from October 2015 to February 2016. The study was conducted by physicians in compliance with the Indian Good Clinical Practice Guidelines, and the Ethical Principles of the Declaration of Helsinki. The study was approved by the Office of the Drug Controller General of India, and was registered with the Clinical Trials Registry of India (www.ctri.nic.in; CTRI/2015/09/006209). The study was initiated after review and approval by the Institutional Ethics Committees at each of the seven participating study centers. Written informed consent was taken from each subject, and the entire process of informed consent was video recorded, as per the regulatory requirements of the country.

### Subjects

Subjects of either sex, ≥18 years of age, attending the outpatient department for influenza vaccination and having adequate literacy to complete the diary cards were enrolled into the clinical trial. Subjects were excluded from the study if they had any past history of hypersensitivity reaction, neurological disorder (Guillain–Barré syndrome or others) or any serious adverse event to any component of influenza vaccine, egg, chicken proteins, aminoglycoside antibiotics; history of administration of any influenza vaccine within the past 6 months or history of laboratory confirmed influenza in the past. Other exclusion criteria were subjects with thrombocytopenia or any coagulation disorder, or subjects on anticoagulation therapy; subjects with confirmed or suspected immunosuppressive or immunodeficiency disorder, or subjects on any immunosuppressive or immunostimulant therapy; subjects with any clinically significant systemic disorder such as cardiovascular, respiratory, neurologic, gastrointestinal, hepatic, renal, endocrine, hematological or immunological disorder; subjects with febrile illness (temperature ≥ 100.4°F) at the time of enrolment, or acute respiratory pathology or infections requiring systemic antibiotic or antiviral therapy during the preceding 7 days; subjects administered blood, blood containing products or immunoglobulins within the last 3 months or planned administration during the study; subjects with history of any other vaccine administration within the last 30 days or planned to be administered during the study period; pregnant and lactating women & female subjects not using acceptable contraceptive measures (double barrier methods, oral or injectable hormonal contraceptives or surgical sterilization); and subjects who had participated in another clinical trial in the past 3 months. Subjects were permitted to use any medication for the treatment of concomitant diseases or adverse events during the study period that were not known to interact with the immunogenicity of the vaccine. However, a record of the same was maintained in the Case Record Form.

### Study procedures and vaccines

Subjects satisfying the eligibility criteria were randomized in a 1:1 ratio, as per a centralized computer generated randomization schedule, to receive either the TetIV (M/s Cadila Healthcare India) or the TriIV (Sanofi India Private Limited). As tetravalent influenza vaccine is not licensed for marketing in India, a licensed Trivalent Influenza vaccine was used as a comparator. As the trial was conducted in 2015–2016, both the vaccines used in the study complied with the WHO recommendation for influenza vaccines for use in the 2015–2016 influenza season (Northern Hemisphere). The TetIV contained ≥15 μg of A/California/7/2009/(H1N1)-Like Virus; ≥15 μg of A/Switzerland/9715293/2013/H3N2-Like Virus; ≥15 μg of B/Phuket/3073/2013-Like Virus (B/Yamagata) and ≥15 μg of B/Brisbane/60/2008-Like Virus (B/Victoria) in 0.5 ml of PBS; while the Sanofi TriIV contained 15 μg of A/California/7/2009/(H1N1)-Like Strain; 15 μg of A/Switzerland/9715293/2013/H3N2-Like Strain and 15 μg of B/Phuket/3073/2013-Like Strain (B/Yamagata). All the subjects were administered a 0.5 mL single dose of the vaccine in a single blind manner as intramuscular injection in the deltoid muscle in the upper arm taking aseptic precautions. A double blind study was not planned as there was difference in the formulation of the two study vaccines (TetIV in a vial and TriIV in a pre-filled syringe). All the subjects were monitored for adverse events for at least 30 minutes following vaccination. Thereafter, the subjects were monitored for 42 d on an outpatient basis, with scheduled visits on post-vaccination days 7, 21, and 42.

### Immunogenicity and safety assessments

Ten milliliter of blood was collected from the subjects before vaccination and 21 d after vaccination for assessment of serum antibody against the vaccine strains of A/H1N1, A/H3N2, B/Yamagata (B/Phuket) and B/Victoria (B/Brisbane) by a validated haemaglutination inhibition assay. The assay utilized chicken red blood cells (RBCs) and aserum-virus incubation at room temperature (25°C) to provide optimal sensitivity and specificity for the vaccine antigens. Assays were performed at Cliantha Research Limited, India by laboratory scientists who were blinded to vaccine assignment. Controls (including serum control, RBC control and antigen control) and participant sera were incubated with neuraminidase to eliminate non-specific inhibitors. Spontaneous anti-species agglutinins were adsorbed by incubating the sera with a suspension of chicken RBCs. Ten two-fold dilutions (starting at 1:10) of the treated sera were incubated with a previously titrated influenza virus solution at a concentration of 4 haemagglutination units/25µL. Following incubation, the results of the assay were read, with the endpoint being the highest serum dilution in which complete inhibition of haemagglutination occurred. Both the pre-vaccination and post-vaccination sera of all the subjects were tested in the same assay and on the same plate. All the serum samples were tested in duplicate.

Seroconversion rate was defined as the percentage of subjects with either a pre-vaccination HI titer <1:10 and a post-vaccination HI titer ≥1:40 or a pre-vaccination HI titer ≥1:10 and a minimum four-fold rise in post-vaccination HI antibody titer. Seroprotection rate was defined as proportion of subjects with post-vaccination titer ≥1:40. Geometric mean titre (GMT) of serum antibody against the vaccine strains of A/H1N1, A/H3N2, B/Victoria and B/Yamagata were assessed at baseline (prior to vaccination) and at 21 d post-vaccination. For the purpose of calculation, any antibody titre <10 (undetectable) was expressed as 5.

Diary cards were provided to the enrolled subjects to record solicited adverse events including pain, tenderness, redness, swelling, fever, nausea, vomiting, headache, myalgia, fatigue and joint pain for 7 d following vaccination. All the other adverse events recorded during the first week after vaccination, or any adverse event noted after the first week, were recorded as unsolicited adverse events. The adverse events were graded from grade 1 to grade 4 based on criteria defined in ‘Guidance for Industry – Toxicity Grading Scale for Healthy Adult and Adolescent Volunteers Enrolled in Preventive Vaccine Clinical Trials, 2007'.^18^

### Statistical analysis

The primary objective of this study was to demonstrate that the lower boundary of the two-sided 95% confidence interval for the seroconversion and the seroprotection rate for all the four strains as calculated 21 d after vaccination in the TetIV group exceeded 40% and 70%, respectively. This was adapted from the CHMP,^7^ US FDA^8^ and WHO^9^ guidance on the requirements of clinical data for licensure of seasonal inactivated influenza vaccines. The seroconversion rate and the seroprotection rate for all the four strains as obtained with the test vaccine were compared with that of the reference vaccine. The geometric mean titres for all the four strains, 21 d after vaccination were also compared between the two groups. The seroconversion rate, seroprotection rate and geometric mean titres in the two groups stratified by age groups (adults: ≥18 to 60 years; and elderly: >60 years) were also calculated.

Assuming the true seroconversion rate to be at least 50% and true seroprotection rate to be at least 80% for each of the four strains, a sample size of 175 subjects provided a power of more than 99% to achieve the primary objective. The seroconversion rate and seroprotection rate in the two groups were compared using chi-square tests. Unpaired t-test was used to compare the log-transformed data of the antibody titers of the two groups. *P* values < 0.05 were considered statistically significant.

Immunogenicity assessments were done both for the Per Protocol (PP) Population (defined as all randomized subjects who had completed the trial with no violations, as per the protocol, with both pre- and post-vaccination immunological assessments) and the modified Intention to Treat (mITT) Population (defined as all the randomized subjects with both pre- and post-vaccination immunological assessments, including subjects with protocol violations). The PP analysis was considered definitive and has been presented in the results section. The safety population included all subjects who were administered the study vaccine and had been available for a 30 minute observation period for safety assessment.

## References

[1] Patria MF, Tagliabue C, Longhi B, Esposito S Influenza vaccination in children at high risk of respiratory disease. Ther Adv Vaccines. 2013 5;1(1):21–31. doi: 10.1177/2051013613480770. 24757513 PMC3967668

[2] Nichol KL, D'Heilly SJ, Greenberg ME, Ehlinger E Burden of influenza-like illness and effectiveness of influenza vaccination among working adults aged 50–64 years. Clin Infect Dis. 2009;48(3):292–8. doi:10.1086/595842. PMID:19115970 19115970

[3] Belshe RB The need for quadrivalent vaccine against seasonal influenza. Vaccine 2010;28(September (Suppl. 4)):D45–53. doi:10.1016/j.vaccine.2010.08.028. PMID:20713260 20713260

[4] Reed C, Meltzer MI, Finelli L, Fiore A Public health impact of including two lineages of influenza B in a quadrivalent seasonal influenza vaccine. Vaccine 2012;30(March (11)):1993–8. doi:10.1016/j.vaccine.2011.12.098. PMID:22226861 22226861

[5] World Health Organization Recommended composition of influenza virus vaccines for use in the 2012–2013 northern hemisphere influenza season; 2012, http://www.who.int/influenza/vaccines/virus/recommendations/201202_recommendation.pdf?ua=1 (accessed 095, 2017)

[6] Data on file

[7] Committee for Medicinal Products for Human Use Note for guidance on harmonisation of requirements for influenza vaccines. CPMP/BWP/214/96 12 March 1997 http://www.ema.europa.eu/docs/en_GB/document_library/Scientific_guideline/2009/09/WC500003945.pdf (accessed 095, 2017)

[8] US Food and Drug Administration Guidance for Industry Clinical Data Needed to Support the Licensure of Seasonal Inactivated Influenza Vaccines; May 2007, https://www.fda.gov/downloads/BiologicsBloodVaccines/GuidanceComplianceRegulatoryInformation/Guidances/Vaccines/ucm091990.pdf (accessed 095, 2017)

[9] World health Organization Guidelines on regulatory preparedness for human pandemic influenza vaccines (Adopted 2007). WHO Technical Report Series No. 963, 2011 http://www.who.int/biologicals/vaccines/Annex_2_WHO_TRS_963-3.pdf (accessed 095, 2017)

[10] Kieninger D, Sheldon E, Lin WY, Yu CJ, Bayas JM, Gabor JJ, et al. Immunogenicity, reactogenicity and safety of an inactivated quadrivalent influenza vaccine candidate versus inactivated trivalent influenza vaccine: a phase III, randomized trial in adults aged ≥18 years. BMC Infect. Dis. 2013;13:343. doi:10.1186/1471-2334-13-343. PMID:23883186 23883186 PMC3750613

[11] Ambrose CS, Levin MJ The rationale for quadrivalent influenza vaccines. Hum Vaccin Immunother. 2012 Jan;8(1):81–8. doi:10.4161/hv.8.1.17623. 22252006 PMC3350141

[12] Greenberg DP, Robertson CA, Noss MJ, Blatter MM, Biedenbender R, Decker MD Safety and immunogenicity of a quadrivalent inactivated influenza vaccine compared to licensed trivalent inactivated influenza vaccines in adults. Vaccine. 2013;31(5):770–6. doi:10.1016/j.vaccine.2012.11.074. PMID:23228813 23228813

[13] Camilloni B, Neri M, Lepri E, Basileo M, Sigismondi N, Puzelli S, Donatelli I, Iorio AM An influenza B outbreak during the 2007/2008 winter among appropriately immunized elderly people living in a nursing home. Vaccine 2012;28(47):7536–41 doi:10.1016/j.vaccine.2010.08.064. 20846530

[14] Song JY, Cheong HJ, Hwang IS, Choi WS, Jo YM, Park DW, et al. Long-term immunogenicity of influenza vaccine among the elderly: risk factors for poor immune response and persistence. Vaccine 2010;28(May (23)):3929–35. doi:10.1016/j.vaccine.2010.03.067. PMID:20394719 20394719

[15] Tinoco JC, Pavia-Ruz N, Cruz-Valdez A, Aranza Doniz C, Chandrasekaran V, Dewé W, Liu A, Innis BL, Jain VK Immunogenicity, reactogenicity, and safety of inactivated quadrivalent influenza vaccine candidate versus inactivated trivalent influenza vaccine in healthy adults aged ≥18 years: a phase III, randomized trial. Vaccine. 2014 Mar 14;32(13):1480–7. doi:10.1016/j.vaccine.2014.01.022. 24486352

[16] Influenza vaccine – Fluzone quadrivalent [package insert]. Swiftwater: Sanofi Pasteur

[17] Influenza vaccine – Fluarix quadrivalent [package insert]. Germany: GlaxoSmithKline Biologicals.

[18] US Food and Drug Administration Guidance for industry: Toxicity grading scale for healthy adult and adolescent volunteers enrolled in preventive vaccine clinical trials. 2007 Available at http://www.fda.gov/BiologicsBloodVaccines/GuidanceComplianceRegulatoryInformation/Guidances/Vaccines/ucm074775.htm (last accessed 2592017.

